# Correlation between the geographical origin of *Helicobacter pylori homB*-positive strains and their clinical outcomes: a systematic review and meta-analysis

**DOI:** 10.1186/s12876-021-01764-y

**Published:** 2021-04-20

**Authors:** Masoud Keikha, Mohsen Karbalaei

**Affiliations:** 1 Department of Microbiology and Virology, Faculty of Medicine, Mashhad University of Medical Sciences, Mashhad, Iran; 2 Student Research Committee, Mashhad University of Medical Sciences, Mashhad, Iran; 3 Department of Microbiology and Virology, School of Medicine, Jiroft University of Medical Sciences, Jiroft, Iran

**Keywords:** Gastric cancer, *Helicobacter pylori*, *HomB* gene, MALT, Peptic ulcer

## Abstract

**Background:**

In general, all virulence factors of *Helicobacter pylori* (*H.* pylori) are involved in its infections. However, recent studies have shown that the *homB* gene is one of the virulence genes that affects the severity of the clinical results of this bacterium.

**Methods:**

The main purpose of this study was to investigate the relationship between the presence of *homB* gene in *H. pylori* and the progression of its infection to peptic ulcer and gastric cancer. In the present study, we conducted a systematic search to collect all articles related to the effect of *homB*-positive strains on clinical outcomes. Finally, 12 eligible studies according to our criteria were included in this meta-analysis and the effect of *homB* gene on gastric ulcer and gastric cancer diseases was evaluated by summary odds ratio (OR).

**Results:**

Current results showed that the *homB*-positive strains significantly increase the risk of peptic ulcer (OR 1.36; 1.07–1.72 with 95% CIs), especially in western countries (OR 1.61; 1.20–2.14 with 95% CIs). Moreover, we observed a positive association between the *homB* gene and risk of gastric cancer (OR 2.16; 1.37–3.40 with 95% CIs). In addition, based on subgroup analysis, it was found that the presence of this gene in *H. pylori* strains increases the risk of gastric cancer in the Asian population (OR 3.71; 1.85–7.45 with 95% CIs).

**Conclusions:**

Overall, in the present study we found that *homB* gene is responsible for the progressing of primary infection to severe complications, in particular peptic ulcer in western countries and gastric cancer in Asian countries.

## Background

*Helicobacter pylori* (*H. pylori*), formerly known as *Campylobacter pyloridis*, is a gram-negative, microaerophilic, helical, and motile (lophotrichous flagella) bacterium that colonizes the gastric sub-mucosa of more than 50% of the world’s population [1]. Infection with this pathogen generally occurs in childhood and may continue asymptomatic for life [2]. However in 15–20% of infected people, the primary infection progresses to worse conditions such as peptic ulcer disease (PUD), duodenal ulcer (DU), gastric ulcer (GU), gastric adenocarcinoma, and mucosa-associated lymphoid tissue (MALT) lymphoma; PUD rarely occurs in children [3–5]. According to the literature, interactions between bacteria, the host genome, and environmental conditions play a decisive role in the development of primary infection to severe clinical outcomes [6]. Although the main role of some virulence genes such as *vacA* and *cagA* is well known, the effect of other virulence factors on bacterial pathogenesis is unclear and needs further study [7–10]. The *H. pylori* genome encodes about 1,100 genes, of which 500–600 are strain-specific genes and in turn contribute in various clinical outcomes [11, 12]. Outer membrane proteins (OMPs) are among the most divergent proteins in this bacterium, encoded by 4–5% of the bacterial genome [13]. The *hom* genes are known as a small paralogous family of adhesion proteins and are distinguished from other OMPs by the signal sequence and hydrophobic motif located in the C-terminal domain [14]. The *hom* family consists of four classes *homA*, *homB*, *homC*, and *homD*, so that *homA* and *homB* are encoded by one locus, while each of *homC* and *homD* is encoded by a distinct locus [14, 15]. Despite 90% similarity between *homA* and *homB*, studies show that the distribution of each is specific in each geographic area, so that *homA* is a diagnostic marker for East Asian strains, and *homB* has global distribution [15, 16]. Recently, the relationship between *homB* and severe clinical outcomes has attracted much attention; the product of *homB*, HomB is a virulence factor that contributes to several bacterial activities such as biofilm formation, antibiotic resistance, delivery of CagA from type 4 secretion system (T4SS), induction of IL-8 production, gastritis, corpus atrophy, and persistent colonization [17–20]. Oleastro et al. first showed a significant association between the *homb*-positive strains and the progression of infection to PUD in Portuguese children [21]. In another study, they found that the ability of *homB* knockout mutant strains to bind to gastric epithelium was significantly reduced compared to *homB*-positive strains [22]. Jung et al. found a positive correlation between the simultaneous presence of *cagA* and *homB* genes in East Asian strains; they showed that having two copies of *homB* gene could increase the risk of PUD [23]. In contrast, the *homA* gene is correlated with non-ulcer dyspepsia (NUD), in other words, it appears that there is no significant relationship between *homA* and gastritis and corpus atrophy [16, 19]. In the present meta-analysis, we evaluated the association between *homB*-positive strains of *H. pylori* and several clinical outcomes.

## Methods

### Literature search strategy

At the first, a systematic search was performed using global databases such as Scopus, Web of Science, and PubMed to collect all the studies relevant to our purpose. All selective studies were related to the association between *homB* and clinical outcomes such as PUD, duodenal ulcer, gastric ulcer, and gastric cancer (GC). In this study, articles published up to December 2020 were retrieved separately by two authors (MK1 and MK2). Search terms were selected based on MeSH thesaurus including “*Helicobacter pylori*”, “*H. pylori*”, and “*homB*”; articles were search regardless of publication date and language.

### Inclusion criteria

In this step, we used the full-text of all published case–control studies examining the relationship between the *homB* and severe clinical outcomes (patients with gastritis and NUD were considered as control group, while patients with PUD, GU, DU, and GC were assumed as case group). The inclusion criteria in adult populations included; (1) published researches on the presence of *homB* confirmed by polymerase chain reaction (PCR) or immunoblotting, (2) exploratory studies on the association between *H. pylori homB* gene and severe clinical outcomes, (3) articles published in English, and (4) articles about human adults. All relevant documents were independently reviewed by two authors (MK1 and MK2).

### Exclusion criteria

Exclusion criteria included; (1) duplicate articles, (2) studies without raw data, (3) studies with control groups only (gastritis and NUD), (4) articles with repetitive samples and results, (5) conference abstracts, review articles, and case series, (6) in vitro or animal experiments, (7) articles with insufficient data for calculating OR with 95% confidence intervals (CIs), (8) studies on non-*homB* gene, and 9) (studies conducted in child populations.

### Quality assessment and data extraction

Using the Newcastle–Ottawa Scale (NOS), 12 eligible studies were selected [21–32]. Required information such as first author, year of publication, country, population sample size, number of *H.pylori* strains, Frequency of *homB* in strains creating different clinical futures, diagnostic methods and reference number are listed in Table 1.

**Table 1:** Characteristics of included studies

First author	Publication year	Country	Population size	*H*. *pylori* strains	*homB* expressing *H*. *pylori* strains					Diagnostic method	Refs.
					Gastritis or NUD	PUD	DU	GU	GC		
Oleastro et al.	2006	Portugal	45	45	11	12	NA	NA	NA	Culture-PCR	19
Oleastro et al.	2008	Portugal	190	190	18	25	NA	NA	NA	Culture-PCR	20
Jung et al.	2009	Japan	286	286	79	50	50	NA	46	Culture-PCR	21
Oleastro et al.	2009	Portugal	372	372	113	150	NA	NA	NA	Culture-PCR	22
Oleastro et al.	2010	Portugal	117	117	21	41	NA	NA	NA	Culture-PCR	23
Hussein et al.	2011	Iraq	134	126	25	15	NA	NA	NA	Culture-PCR	24
Abadi et al.	2011	Iran	138	138	15	13	NA	NA	32	Culture-PCR	25
Khamis et al.	2018	Iraq	471	194	47	42	NA	NA	72	Culture-PCR	26
Šterbenc et al.	2018	Slovenia	343	285	106	3	NA	NA	NA	RUT-PCR	7
Casarotto et al.	2019	Italy	340	53	NA	NA	NA	NA	1.11 (0.31 − 3.91)	Culture-PCR	28
YJlmaz et al.	2019	Turkey	214	82	2	1	NA	1	NA	Culture-PCR	29
Haddadi et al.	2020	Iran	280	128	10	27	NA	NA	9	Culture-PCR	30

### Statistical analysis

All statistical analysis were performed using Comprehensive Meta-Analysis (CMA) software version 2.2 (Biostat, Englewood, NJ, USA). The relationship between *homB* and clinical outcomes was estimated according to the summary odds ratio with 95% CIs. Heterogeneity between studies was assessed through parameters such as *I*^*2*^ index and Cochrane Q test, so that in cases of high heterogeneity (*I *> 25% and Cochrane Q test *p* value > 0.05) and non-significant heterogeneity, we used from random-effects model, and fixed-effects model, respectively. Finally, the publication bias of selected studies was measured using funnel plot asymmetry, Egger’s *p* value, and Begg’s *p* value test [33].

## Results

### Characteristics of selected studies

**Fig. 1:**
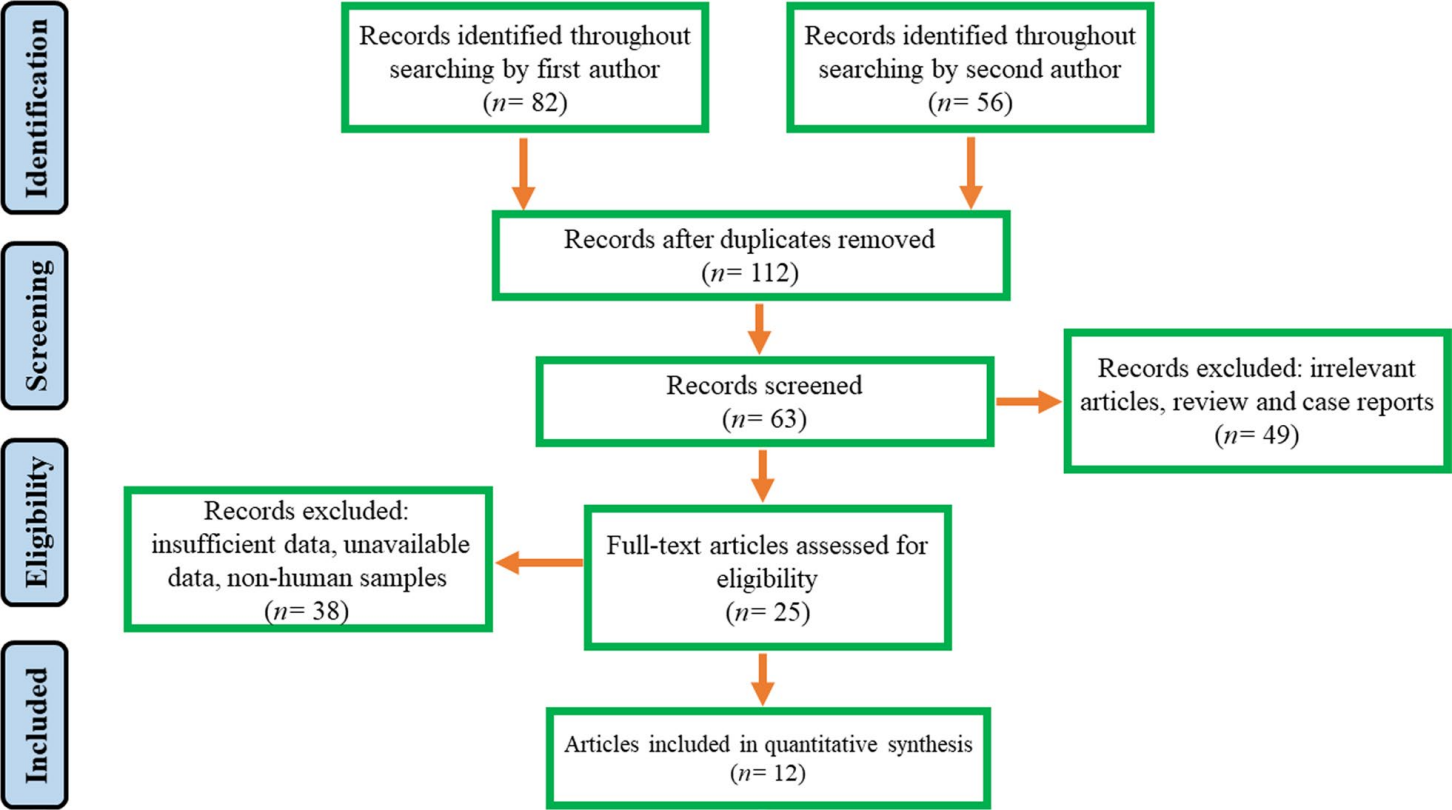
The flowchart of study selection and included studies

Following the initial systematic search, 138 related articles were collected and 126 articles were deleted according to inclusion criteria. The details of comprehensive search processing and study selection are summarized in Fig. 1. In the screening phase, we removed the irrelevant articles such as articles with unclear results and articles that did not meet our criteria. In total, out of 12 studies that met our inclusion criteria, 2930 patients and 2016 strains of *H. pylori* were evaluated. Of these, two studies compared the relationship between *homB* and clinical outcomes in both Western and Asian countries [24, 26], Six studies have been conducted on Western countries [21, 22, 25, 29–31], as well as four studies on the population of Asia [23, 27, 28, 32] (Table 1). Final results of some of eligible studies were contradictory and varied [22, 24, 26, 27].

### Association between homB and PUD

**Fig. 2:**
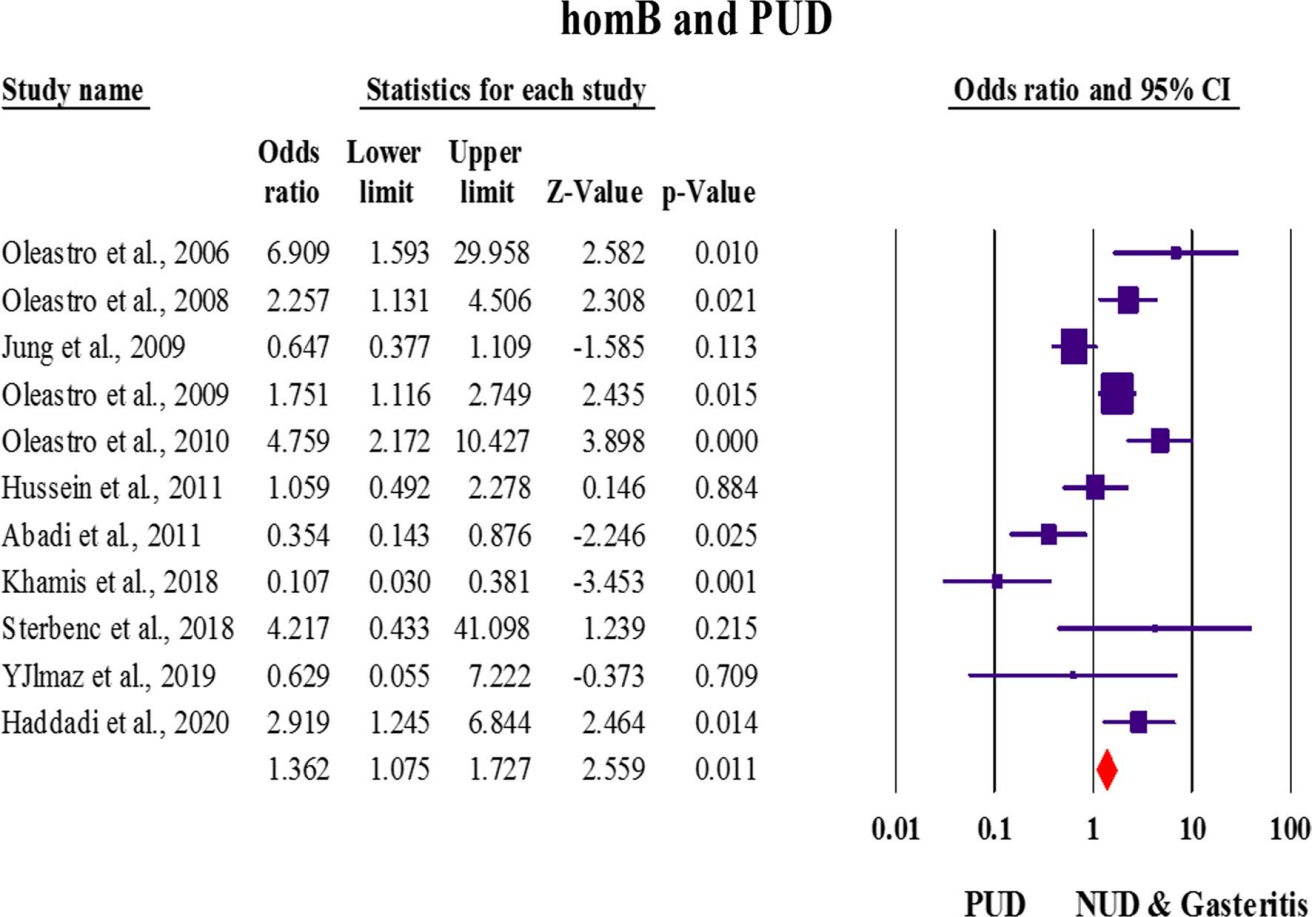
Random-effects meta-analysis forest plot of the OR of peptic ulceration according to *homB*-positive *H. pylori* infection in eleven included studies

With the exception of the study by Casarotto et al., 11 studies had investigated the relationship between *homB* and PUD. The prevalence of *homB* in patients with GC and PUD was estimated at 54.4% [40.8–67.4 with 95% CIs; Effect size (with 95% CIs): 0.59 (0.18–1.00); *I*^*2*^: 96.1; Q-Value: 119.94; *p* value: 0.01; Begg’s *p* value: 0.21; Egger’s *p* value: 0.43]. However, the frequency of *homB* in patients with gastritis/NUD was estimated to be approximately 39.7% [27.6–53.2 with 95% CIs; Effect size (with 95% CIs): 0.43 (0.12–0.75); *p* value: 0.01; Begg’s *p* value: 0.26; Egger’s *p* value: 0.40]. We found that there was a significant relationship between the expression of *homB* gene and the progression of infection to PUD [OR: 1.36; 1.07–1.72 with 95% CIs; *p* value: 0.01; Effect size (with 95% CIs): 0.75 (0.28–1.38); *p* value: 0.01; *I*^*2*^: 81.41; Q-Value: 53.86; *p* value: 0.01; Begg’s *p* value: 0.87; Egger’s *p* value: 0.93]. According to the information, infection with *homB*-expressing strains appears to increase the risk of PUD (Fig. 2). Due to the high heterogeneity between studies, we used subgroup analysis to determine the role of *homB* in the development of primary infection to PUD in Western and Asian countries. Interestingly, a positive relationship was observed between the presence of *homB* gene and PUD in Western countries [OR: 1.61; 1.20–2.14 with 95% CIs; *p* value: 0.01; Effect size (with 95% CIs): 0.88 (0.44–1.43); *p* value: 0.01; *I*^*2*^: 71.65; Q-Value: 24.69; *p* value: 0.01; Begg’s *p* value: 0.90; Egger’s *p* value: 0.43] whereas, there was no meaningful relationship between this gene and PUD in Asian countries [OR: 0.89; 0.57–1.40 with 95% CIs; *p* value: 0.63; Effect size (95%CIs): 0.49 (0.23–0.65); *p* value: 0.01; *I*^*2*^: 83.60; Q-Value: 24.4; *p* value: 0.01; Begg’s *p* value: 0.11; Egger’s *p* value: 0.24]. Lack of access to raw data led to non-assessment the relationship between *homB* and duodenal and gastric ulcers.

### Association between homB and GC

**Fig. 3:**
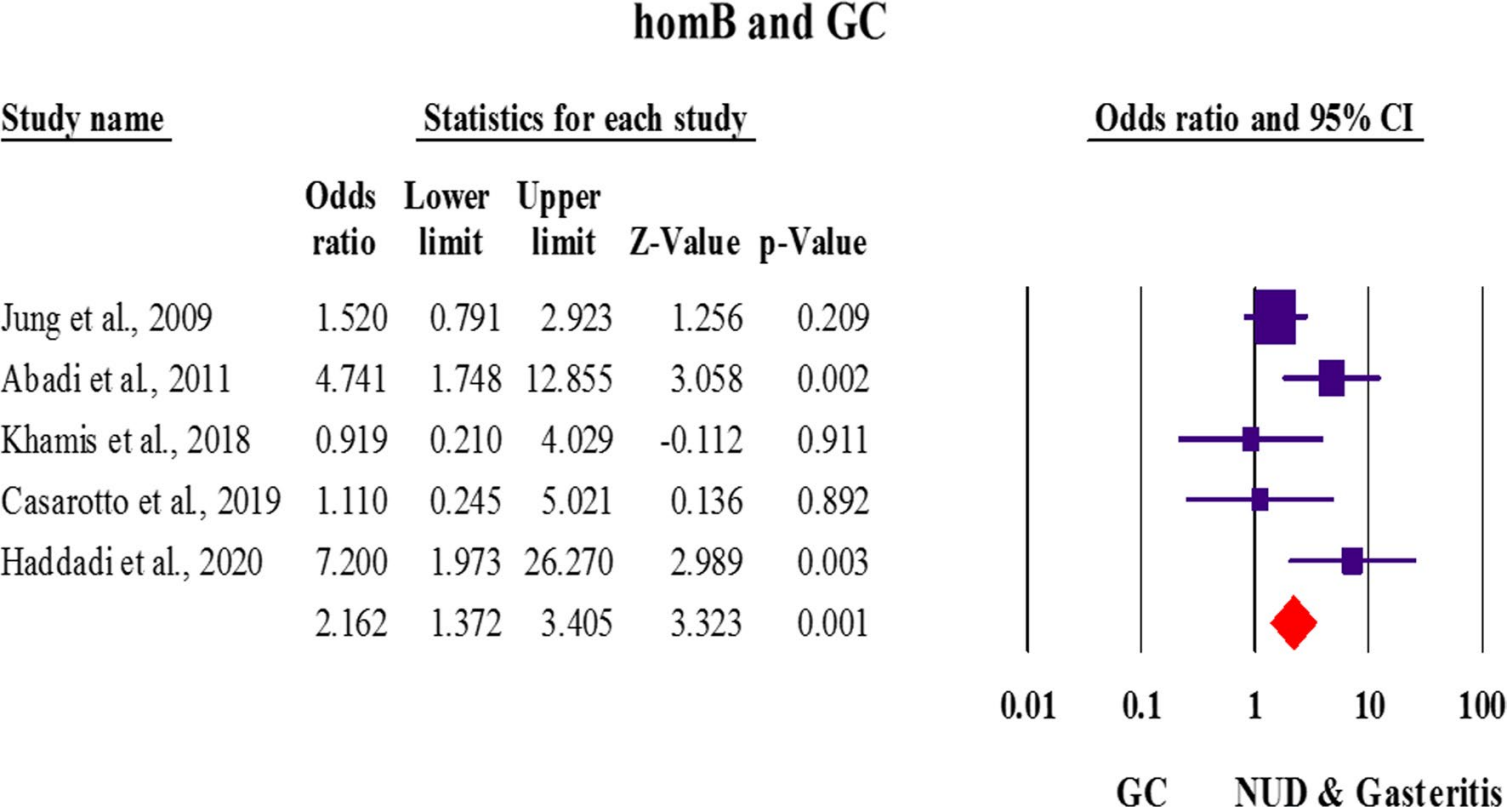
Random-effects meta-analysis forest plot of the OR of gastric adenocarcinoma according to *homB*-positive *H. pylori* infection in five included studies

Five articles had evaluated the relationship between the *homB* gene and incidence of GC (low sample size), however, a positive association was observed between *homB* and GC [OR: 2.16; 1.37–3.40 with 95% CIs; *p* value: 0.01; Effect size (with 95% CIs): 0.54 (0.32–0.77); *p* value: 0.01; *I*^*2*^: 56.29; Q-Value: 9.15; *p* value: 0.05; Begg’s *p* value: 0.80; Egger’s *p* value: 0.77]. The summary of OR showed that the presence of *homB* gene significantly increases the incidence of GC (Fig. 3). In the subgroup analysis process, a positive relationship was observed between *homB*-positive strains and the risk of GC in Asian countries [OR: 3.71; 1.85–7.45 with 95% CIs; *p* value: 0.01; Effect size (with 95% CIs): 2.04 (0.33–3.72); *p* value: 0.01; *I*^*2*^: 57.12; Q-Value: 4.66; *p* value: 0.09]. Although a weak positive relationship was also observed between this gene and the incidence of GC in Western countries, but the threshold was not significant [OR: 1.42; 0.79–2.54 with 95% CIs; *p* value: 0.23; Effect size (with 95%CIs): 0.78 (0.27–0.83); *p* value: 0.01; *I*^*2*^: 0.00; Q-Value: 0.18; *p* value: 0.66]. Regarding the small number of included studies, many studies are needed to find the full relationship between *homB* gene and incidence of GC in patients infected with *H. pylori*.

### Publication bias analysis

Publication bias was estimated based on both Begg’s *p* value and Egger’s *p* value tests, although no significant publication bias was observed. However, funnel plot asymmetry indicated a slight publication bias in the current meta-analysis.

## Discussion

*H. pylori* is one of the most successful pathogens that colonizes the stomach of half the world's population. This bacterium can cause serious clinical consequences such as chronic gastritis, PUD, gastric atrophy and GC [34, 35]. According to documents, approximately 63% of GC cases worldwide are caused by *H. pylori* infection, and the bacterium is also responsible for 75% of gastric ulcers and 90% of duodenal ulcers [27]. The strains of this bacterium are genetically diverse and harbor different virulence genes [36, 37]. Studies in recent decades have shown that these genes are strain-specific (e.g. *vacA*, *cagA*, and *omp*) and play an important role in the immunopathogenesis of *H. pylori* and in the development of serious clinical outcomes [13, 17, 22, 27, 32, 38]. In several studies, the role of the *homB* gene in the pathogenesis of this pathogen was controversial; difference in results are related to differences in diet, environmental condition, hygiene status, age, socioeconomic level, and low sample size [29, 39]. Nevertheless, in the present study, we conducted a comprehensive literature review to assess the role of *homB* in the progression of primary infection to PUD and GC in Western and Asian countries. Oleastro et al. in their study showed that the presence of the *homB* gene is significantly higher than the *homA* gene in Portuguese children with PUD; “on” genotypes consistent *cagA*/*vacAs1*/ *hopQI*/*oipA/homB* strongly were associated with PU disease in children under four years of age [22]. In contrast, in studies on populations of Iraq, Turkey, and South Korea, none of *homA* and *homB* genes were correlated with PU disease [26, 40]. Interestingly, all studies in Western children have shown that the *homB* gene is associated with PUD, while the *homA* gene is more prevalent in the NUD [21, 22, 25]. In present study, frequency of *homB* gene in patients with affected to PUD and GC, severe clinical outcomes significantly was more prevalent than gastritis/NUD cases (54.4% and 39.7%, respectively). In addition, the summary OR showed that there was a significant relationship between *homB*-positive genotype and progression to PUD, especially in Western countries (OR: 1.16; 1.20–2.14 with 95% CIs; *p* value: 0.01), while in Asian countries there was no such relationship (OR: 0.89; 0.57–1.40 with 95% CIs; *p* value: 0.01). Therefore, our findings confirmed the results of previous studies. Also, strains isolated from Western countries contained two copies of the *homB* gene, but most infectious strains in Asian countries had only one copy of each of the *homA* and *homB* genes [19, 22, 31]. Related articles showed that the number of OMP copies also affects the status of bacterial compatibility and plays a role in the formation of clinical outcomes [14, 41, 42]. Recently, the role of *homB* as a cofactor in the increase of gastric adenocarcinoma in Asian countries has attracted much attention. The *homB* gene enhances the attachment of *H. pylori* to gastric epithelium, leading to dysregulation of normal signaling pathways and genetic instability [27, 40]. In addition, this gene increases the risk of GC through interferences such as inducing the inflammatory response, persistent infection, and gastric atrophy [23]. Abadi et al. showed that 78% of the strains isolated from GC patients contained the *homB* gene [27]. Jung et al. found that *cagA*-independent *homB* was associated with GC in Western countries [23]. However, in a study on the Chinese population, despite the presence of the *homB* gene in all isolated strains in patients with PUD and GC, no significant correlation was observed [39]. According to our results, a strong correlation was observed between the *homB* gene and the risk of GC in the Asian population (OR: 3.71; 1.85–7.45 with 95% CIs; *p* value: 0.01), whereas this correlation did not exist in Western countries (OR: 1.42; 0.79–2.54 with 95% CIs; *p* value: 0.66). Thus, depending on the geographical area, the *homB* gene appears to lead to PUD and GC in Western and Asian countries respectively. In several studies, correlation between *homB* and other virulence factors, especially *vacA*, *cagA*, *oipA*, *hopQI*, and *babA* in patients with PUD and GC was investigated. Sterbenc et al. in their study observed that there was no significant difference in the histopathological characteristics of PUD in both groups of children with and without the genotype profiles *vacA*s1m1*/cagA/babA2/hompB* [29]. Similar to this study, Oleastro et al. found that *homB*, independent of the *cagA* + /*vacA*s1 genotype profile, increases PUD risk in Western countries [24]. However, in other studies, it was shown that there is a significant correlation between *homB* and *cagA*, and *homB* also acts as a cofactor in complications such as PUD [23, 27, 40]. Due to the lack of raw data and uncertain results, we could not evaluate the relationship between *homB* and *cagA* in patients with PUD, but in GC cases, a weak correlation was observed (OR: 1.47; 0.95–2.28 with 95% CIs; *p* value: 0.79; I2: 80.15; Q-Value: 15.1; *p* value: 0.02; Egger’s *p* value: 0.07; Begg’s *p* value: 0.08). In the end it must be said, our study had several limitations such as low sample size, low number of included studies, inaccessibility to raw data, high heterogeneity in some studies, and also slight publication bias based on asymmetry of funnel plot. Hence, we need further studies to confirm the present findings.

## Conclusions

Our results suggest that the presence of the *homB* gene in *H. pylori* strains contributes to the development of primary infection to severe clinical outcomes. We found that the *homB* gene could increase the risk of PUD in Western countries as well as GC in Asian countries.

## Data Availability

All data generated or analyzed during this study are included in this published article and its supplementary information files.

## Abbreviations

*H. pylori*: *Helicobacter pylori*
PUD: Peptic ulcer disease
DU: Duodenal ulcer
GU: Gastric ulcer
GC: Gastric cancer
NUD: Non-ulcer dyspepsia
MALT: Mucosa-associated lymphoid tissue
OMPs: Outer membrane proteins
T4SS: Type 4 secretion system
NUD: Non-ulcer dyspepsia

## References

[1] Marshall B, Warren JR (1984). Unidentified curved bacilli in the stomach of patients with gastritis and peptic ulceration. The lancet.

[2] Axon A (2014). Helicobacter pylori and public health. Helicobacter.

[3] Karbalaei M, Khorshidi M, Sisakht-pour B, Ghazvini K, Farsiani H, Youssefi M, et al. What are the effects of IL-1β (rs1143634), IL-17A promoter (rs2275913) and TLR4 (rs4986790) gene polymorphism on the outcomes of infection with H. pylori within as Iranian population: a systematic review and meta-analysis. Gene Rep. 2020:100735.

[4] Malaty HM, El-Kasabany A, Graham DY, Miller CC, Reddy SG, Srinivasan SR (2002). Age at acquisition of Helicobacter pylori infection: a follow-up study from infancy to adulthood. The Lancet.

[5] Kato S, Nishino Y, Ozawa K, Konno M, Maisawa SI, Toyoda S (2004). The prevalence of Helicobacter pylori in Japanese children with gastritis or peptic ulcer disease. J Gastroenterol..

[6] Keikha M (2020). Is there a relationship between Helicobacter pylori vacA i1 or i2 alleles and development into peptic ulcer and gastric cancer? A meta-analysis study on an Iranian population. New Microbes and New Infections.

[7] Homan M, Luzar B, Kocjan BJ, Mocilnik T, Shrestha M, Kveder M (2009). Prevalence and clinical relevance of cagA, vacA, and iceA genotypes of Helicobacter pylori isolated from Slovenian children. J Pediatr Gastroenterol Nutr.

[8] Yousefi B, Mohammadlou M, Abdollahi M, Salek Farrokhi A, Karbalaei M, Keikha M (2019). Epigenetic changes in gastric cancer induction by Helicobacter pylori. J Cell Physiol.

[9] Yamaoka Y, Graham DY (2014). Helicobacter pylori virulence and cancer pathogenesis. Future Oncol.

[10] Keikha M, Ali-Hassanzadeh M, Karbalaei M (2020). Association of Helicobacter pylori vacA genotypes and peptic ulcer in Iranian population: a systematic review and meta-analysis. BMC Gastroenterol.

[11] Ali A, Naz A, Soares SC, Bakhtiar M, Tiwari S, Hassan SS, et al. Pan-genome analysis of human gastric pathogen *H. pylori*: comparative genomics and pathogenomics approaches to identify regions associated with pathogenicity and prediction of potential core therapeutic targets. BioMed Res Int. 2015;2015. 10.1155/2015/139580 PMC4325212 25705648

[12] Gressmann H, Linz B, Ghai R, Pleissner K-P, Schlapbach R, Yamaoka Y (2005). Gain and loss of multiple genes during the evolution of Helicobacter pylori. PLoS Genet.

[13] Oleastro M, Ménard A (2013). The role of Helicobacter pylori outer membrane proteins in adherence and pathogenesis. Biology.

[14] Alm RA, Bina J, Andrews BM, Doig P, Hancock RE (2000). Comparative genomics of Helicobacter pylori: analysis of the outer membrane protein families. Infect Immun.

[15] Servetas SL, Kim A, Su H, Cha JH, Merrell DS (2018). Comparative analysis of the Hom family of outer membrane proteins in isolates from two geographically distinct regions: the United States and South Korea. Helicobacter.

[16] Oleastro M, Cordeiro R, Ménard A, Gomes JP (2010). Allelic diversity among Helicobacter pylori outer membrane protein genes homB and homA generated by recombination. J Bacteriol.

[17] Servetas SL, Doster RS, Kim A, Windham IH, Cha J-H, Gaddy JA (2018). ArsRS-dependent regulation of homB contributes to Helicobacter pylori biofilm formation. Front Microbiol.

[18] Šterbenc A, Jarc E, Poljak M, Homan M (2019). Helicobacter pylori virulence genes. World J Gastroenterol.

[19] Oleastro M, Cordeiro R, Ménard A, Yamaoka Y, Queiroz D, Mégraud F (2009). Allelic diversity and phylogeny of homB, a novel co-virulence marker of *Helicobacter pylori*. BMC Microbiol.

[20] Keikha M, Eslami M, Yousefi B, Ghasemian A, Karbalaei M (2019). Potential antigen candidates for subunit vaccine development against *Helicobacter pylori* infection. J Cell Physiol.

[21] Oleastro M, Monteiro L, Lehours P, Mégraud F, Ménard A (2006). Identification of markers for *Helicobacter pylori* strains isolated from children with peptic ulcer disease by suppressive subtractive hybridization. Infect Immun.

[22] Oleastro M, Cordeiro R, Ferrand J, Nunes B, Lehours P, Carvalho-Oliveira I (2008). Evaluation of the clinical significance of homb a novel candidate marker of helicobacter pylori strains associated with peptic ulcer disease. J Infect Dis.

[23] Jung SW, Sugimoto M, Graham DY, Yamaoka Y (2009). homB status of Helicobacter pylori as a novel marker to distinguish gastric cancer from duodenal ulcer. J Clin Microbiol.

[24] Oleastro M, Cordeiro R, Yamaoka Y, Queiroz D, Mégraud F, Monteiro L (2009). Disease association with two Helicobacter pylori duplicate outer membrane protein genes, homB and homA. Gut Pathogens.

[25] Oleastro M, Santos A, Cordeiro R, Nunes B, Mégraud F, Ménard A (2010). Clinical relevance and diversity of two homologous genes encoding glycosyltransferases in *Helicobacter pylori*. J Clin Microbiol.

[26] Hussein NR (2011). A study of Helicobacter pylori outer-membrane proteins (hom) A and B in Iraq and Turkey. J Infect Public Health.

[27] Abadi ATB, Rafiei A, Ajami A, Hosseini V, Taghvaei T, Jones KR (2011). Helicobacter pylori homB, but not cagA, is associated with gastric cancer in Iran. J Clin Microbiol.

[28] Khamis AS, Al-Jibouri LF, Al-Marzoqi AH, Shalan AA, Al-Taee ZM, Al Morshdi SF (2018). Helicobacter pylori genotype as predicts risk of (ulcer disease, gastric cancer, non-ulcer dyspepsia); role of some genes mediated signaling in infection. J Pharm Sci Res.

[29] Šterbenc A, Poljak M, Zidar N, Luzar B, Homan M (2018). Prevalence of the Helicobacter pylori homA and homB genes and their correlation with histological parameters in children. Microb Pathog.

[30] Casarotto M, Pratesi C, Bidoli E, Maiero S, Magris R, Steffan A (2019). Differential *Helicobacter pylori* Plasticity in the gastric niche of subjects at increased gastric cancer risk. Pathogens.

[31] Yılmaz N, Koruk Özer M. The prevalence of Helicobacter pylori babA, homB, aspA, and sabA genes and its relationship with clinical outcomes in Turkey. Can J Gastroenterol Hepatol. 2019;2019. 10.1155/2019/1271872 PMC6595381 31312620

[32] Haddadi M-H, Negahdari B, Asadolahi R, Bazargani A. Helicobacter pylori antibiotic resistance and correlation with cagA motifs and homB gene. Postgrad Med. 2020:1–9. 10.1080/00325481.2020.1753406 32281451

[33] Karbalaei M, Keikha M. Potential association between the hopQ alleles of *Helicobacter pylori* and gastrointestinal diseases: a systematic review and meta-analysis. Meta Gene. 2020:100816.

[34] Franceschi F, Zuccalà G, Roccarina D, Gasbarrini A (2014). Clinical effects of Helicobacter pylori outside the stomach. Nat Rev Gastroenterol Hepatol.

[35] Youssefi M, Tafaghodi M, Farsiani H, Ghazvini K, Keikha M. Helicobacter pylori infection and autoimmune diseases; Is there an association with systemic lupus erythematosus, rheumatoid arthritis, autoimmune atrophy gastritis and autoimmune pancreatitis? A systematic review and meta-analysis study. J Microbiol Immunol Infect. 2020. 10.1016/j.jmii.2020.08.011 32891538

[36] Parkin DM (2006). The global health burden of infection-associated cancers in the year 2002. Int J Cancer.

[37] Blaser MJ (1998). Helicobacter pylori and gastric diseases. BMJ.

[38] Safaralizadeh R, Dastmalchi N, Hosseinpourfeizi M, Latifi-Navid S (2017). Helicobacter pylori virulence factors in relation to gastrointestinal diseases in Iran. Microb Pathog.

[39] Zhao Q, Song C, Wang K, Li D, Yang Y, Liu D, et al. Prevalence of Helicobacter pylori babA, oipA, sabA, and homB genes in isolates from Chinese patients with different gastroduodenal diseases. Med Microbiol Immunol. 2020:1–13. 10.1007/s00430-020-00666-2 32219508

[40] Kang J, Jones KR, Jang S, Olsen CH, Yoo Y-J, Merrell DS (2012). The geographic origin of Helicobacter pylori influences the association of the homB gene with gastric cancer. J Clin Microbiol.

[41] Ilver D, Arnqvist A, Ögren J, Frick I-M, Kersulyte D, Incecik ET (1998). Helicobacter pylori adhesin binding fucosylated histo-blood group antigens revealed by retagging. Science.

[42] Solnick JV, Hansen LM, Salama NR, Boonjakuakul JK, Syvanen M (2004). Modification of Helicobacter pylori outer membrane protein expression during experimental infection of rhesus macaques. Proc Natl Acad Sci.

